# Overactive bladder phenotype induced by chronic activation of hypothalamic neuroendocrine stress pathways in rats with no extrinsic behavioral stress applied

**DOI:** 10.1038/s41598-025-32428-6

**Published:** 2025-12-21

**Authors:** Jenan Husain, Alexandra Bakhareva, Anna Pace, Maria Noterman-Soulinthavong, Gerald M. Herrera, Benedek Erdos

**Affiliations:** Department of Pharmacology, University of Vermont, Burlington, 05401 USA

**Keywords:** Overactive bladder, Urinary bladder dysfunction, Stress, Hypothalamus, Neuroscience, Physiology, Urology

## Abstract

Overactive urinary bladder (OAB) negatively impacts quality of life, and stress is known to play a key role in its development. However, the mechanisms linking stress to OAB are not yet fully understood. This study examined how chronic activation of neuroendocrine stress pathways, independently of environmental or psychological stressors, affects bladder function and the control of micturition. Utilizing the central role of brain-derived-neurotrophic factor (BDNF) in orchestrating the neuroendocrine stress response within the paraventricular nucleus of the hypothalamus (PVN), our novel experimental model subjected 10-week-old male Sprague Dawley rats to bilateral PVN injections of AAV2 viral vectors expressing either BDNF or GFP (for control). Urine voiding behavior was assessed in UroVoid metabolic cages over 14 weeks post-injections. Bladder strip myography, assessment of bladder wall mechanics, and histology were also conducted to determine any BDNF-induced differences in bladder contractility, capacity and morphology. Prolonged activation of neuroendocrine stress mechanisms with BDNF overexpression in the PVN significantly reduced intermicturition intervals and voided volumes, lowered bladder capacity, and induced relative bladder wall hypertrophy but had no effect on bladder wall mechanics or detrusor contractility. These results indicate that chronic activation of neuroendocrine stress pathways, even without additional environmental or psychological influences of stress, lead to a significant OAB phenotype and reduced bladder capacity.

**Supplementary Information:**

The online version contains supplementary material available at 10.1038/s41598-025-32428-6.

## Introduction

Overactive bladder (OAB) is characterized by a strong urge to urinate and is often accompanied by increased urinary frequency, nocturia and urinary incontinence^1^. This lower urinary tract disorder is associated with detrusor muscle overactivity and central nervous system dysregulation^1,2^. The global prevalence of OAB is around 20%, with women experiencing it at a higher rate (21.9%) than men (16.1%) and this number is projected to increase over time as life expectancy rises^2,3^. OAB can have a significant negative impact on a person’s quality of life and physical and psychological health^2–4^.

A growing body of clinical evidence links OAB and exacerbation of lower urinary tract symptom severity with stress or stress-associated disorders^2,3,5–10^. People with OAB report experiencing higher moderate to severe anxiety^6,8,10^, depression^8–10^, and perceived stress^5–7,10^. Similarly, experimental findings in various rodent models of psychological stress have shown a causal role of stress exposure in development and enhancement of bladder dysfunction with stressed rodents exhibiting altered voiding behavior, bladder contractility, and bladder wall remodeling^11–19^. Despite accumulating evidence pointing towards stress being detrimental for normal bladder function, the underlying mechanisms behind this association remain elusive and likely reflect a complex interplay between genetic, psychosocial and environmental factors along with stimulation of neuroendocrine stress pathways that together influence neuronal control of bladder function^10,19^.

Normal bladder function results from an intricate balance and integration of autonomic and somatic nervous systems. Any disturbance to this delicate balance may lead to bladder dysfunction or overactivity^12,20–30^. However, one of the reasons that identification of underlying mechanisms involved in stress-induced bladder dysfunction remains difficult is because different types and durations of stressors result in widely varying bladder dysfunction. For instance, male rats subjected to two weeks of mild social stress show increases in micturition frequency whereas male rats going through a more intensified version of the same paradigm exhibit urine retention^17,31^. Moreover, behavioral responses to stressors can differ significantly based on the behavioral stress paradigms being utilized. For example, restraint stress in male rodents leads to either no changes or increases in micturition frequency^12,16,32^ while social stress in male rodents induces urine retention^11,12,16,20,29,31,33,34^. Such disparate findings make identifying underlying causes of stress-induced bladder dysfunction rather challenging. To complicate matters further, some stress paradigms influence rodent scent marking behavior and urine voiding behavior^35,36^, making interpretations of bladder dysfunction even more difficult. Since it is unclear how stress or the stress-associated neuroendocrine and physiological changes impact voiding behavior, it is vital to employ an experimental model that can delineate the effects of prolonged central activation of neuroendocrine stress pathways on bladder function and central regulation of micturition while minimizing variability introduced by behavioral stress paradigms.

The paraventricular nucleus of hypothalamus (PVN) is a heterogenous nucleus that plays a critical role in integration of diverse limbic and medullary inputs to coordinate autonomic, endocrine, cardiovascular and metabolic stress responses^37–39^. There are a number of neuromodulators and neurotransmitters that influence the excitability of PVN neurons^40^ but one key regulator is brain derived neurotrophic factor (BDNF). BDNF can produce short- and long-term adaptative changes throughout the CNS via its actions on excitatory and inhibitory neurotransmitter signaling and its trophic functions promoting dendritic branching and synaptic connections^38,40^. Importantly, BDNF is significantly upregulated in the PVN in response to stressful and hypertensive stimuli^41–43^, and plays a key role in activating the different arms of the neuroendocrine stress response within the PVN, including the sympathetic nervous system, hypothalamic–pituitary-adrenal (HPA) axis and vasopressin (AVP) signaling^41–43^. In addition, BDNF is also involved in metabolic regulation, promoting reductions in food intake and body weight gain^44–46^. The central role BDNF plays in the PVN is highlighted by our previous studies where viral vector-mediated BDNF overexpression in the PVN (the PVN-BDNF model) led to marked elevations in blood pressure, heart rate, body temperature, indices of sympathetic activity, and corticosterone levels compared to controls while also promoting slower weight gain^47–49^. Conversely, inhibition of BDNF signaling in the PVN by overexpressing a dominant negative truncated form of the high affinity tropomyosin receptor kinase B receptor decreased stress-induced elevations in blood pressure, independent of baseline cardiovascular function^50^.

Based on these previous studies from our laboratory, in the present work, we used the PVN-BDNF model to determine how central control of micturition and urinary bladder function are affected by a chronic neuroendocrine stress response without variability arising from behavioral aspects of stress paradigms.

## Results

### Characterization of the PVN-BDNF model

**Fig. 1:**
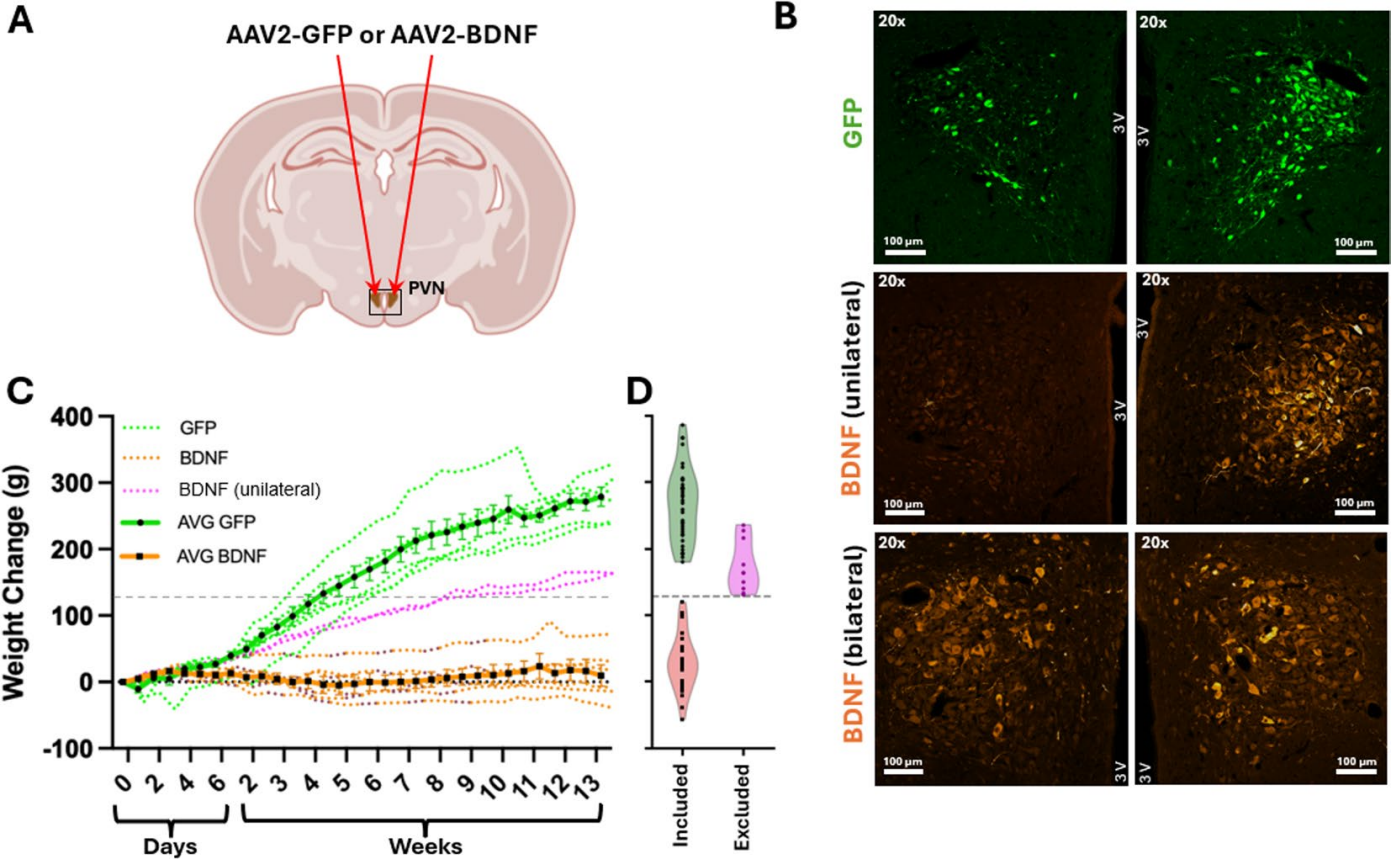
Body weight gain trajectories and verification of gene transduction in rats injected with viral vectors expressing GFP or BDNFmyc within the paraventricular nucleus of hypothalamus (PVN). **A**: Diagram indicating the location of bilateral viral vector injections into the PVN. **B**: Representative fluorescent images of coronal brain sections ~ 1.8 mm posterior to bregma showing bilateral PVN expression of GFP and unilateral or bilateral PVN expressions of BDNFmyc. 3V: third ventricle. **C**: Body weight change of rats involved in immunohistochemical vector verifications. Weight changes from day 0 are expressed as mean ± SEM. Rats with confirmed bilateral PVN expression of BDNFmyc (*n* = 6) showed a markedly reduced weight gain compared to PVN-GFP controls (*n* = 6) over the duration of the study. Body weight trajectories from rats with unilateral PVN expression of BDNFmyc (*n* = 2) are colored in magenta and are not included in the overall PVN-BDNF mean. **D**: Violin plots show distribution of body weight changes for all PVN-GFP (*n* = 38) and PVN-BDNF animals (*n* = 30) included in the study as well as PVN-BDNF rats excluded (*n* = 10) due to missed injections. The horizontal dotted line shows our body weight change cutoff criteria (127.3 g) used to determine if animals were likely to have bilaterial or unilateral PVN-BDNF injections.

To characterize the PVN-BDNF model phenotype and validate the activation of neuroendocrine stress pathways and changes in metabolic regulation, we assessed physiological parameters expected to be affected by BDNF in the PVN. In agreement with previous evidence indicating a key role for BDNF in hypothalamic body weight and metabolic regulation in the hypothalamus^47,51,52^, we found that bilateral BDNF overexpression within the PVN led to significantly lower body weight gains compared to control rats receiving green fluorescent protein (GFP) vector treatment (PVN-GFP rats), and this effect on body weight strongly correlated with the extent of vector-driven BDNF expression (Fig. 1). Figure 1C shows body weight changes in rats in which viral vector injection was verified immunohistochemically (Fig. 1B). Rats with successful bilateral BDNF transduction confirmed by immunofluorescence (*n* = 6) all gained less than 80 g over the 14 weeks following vector injections (Fig. 1C). In contrast, PVN-GFP control rats (*n* = 6) gained greater than 200 g during the same period. Rats in which only unilateral BDNF vector expression was verified by immunofluorescence (*n* = 2) gained more than bilateral PVN-BDNF rats, but less than PVN-GFP rats (Fig. 1C). Since we did not perform immunohistochemical analyses on the brains from all rats used in this study (*n* = 78), we needed a readily available physiological marker to determine if rats were successfully bilaterally injected with BDNF-encoding viral vector or not. Given the striking effect of bilateral BDNF transduction on body weight, we chose body weight change as such a marker.

To achieve a statistically based body weight criterion for determining if a rat was successfully bilaterally injected with BDNF-encoding viral vector, we determined the average weight gain after 14 weeks of the rats with immunohistochemically-verified bilateral BDNF expression (*n* = 6) which was 10 ± 39.1 g (mean ± SD). Assuming a normal distribution of this data, we anticipate that 99.7% of data points will fall within 3 standard deviations of the mean. This would result in an upper edge of 127.3 g. We chose this as our criterion for determining if a rat received successful bilateral BDNF transduction. Figure 1D shows a violin plot of body weight changes after 14 weeks in all 78 rats used in this study. All GFP-injected rats (*n* = 38) were included in the analysis. Thirty of the 40 BNDF-injected rats were included in the study, while 10 of the BDNF rats were excluded from the study because their weight increased by more than 127.3 g (Fig. 1D).

**Fig. 2:**
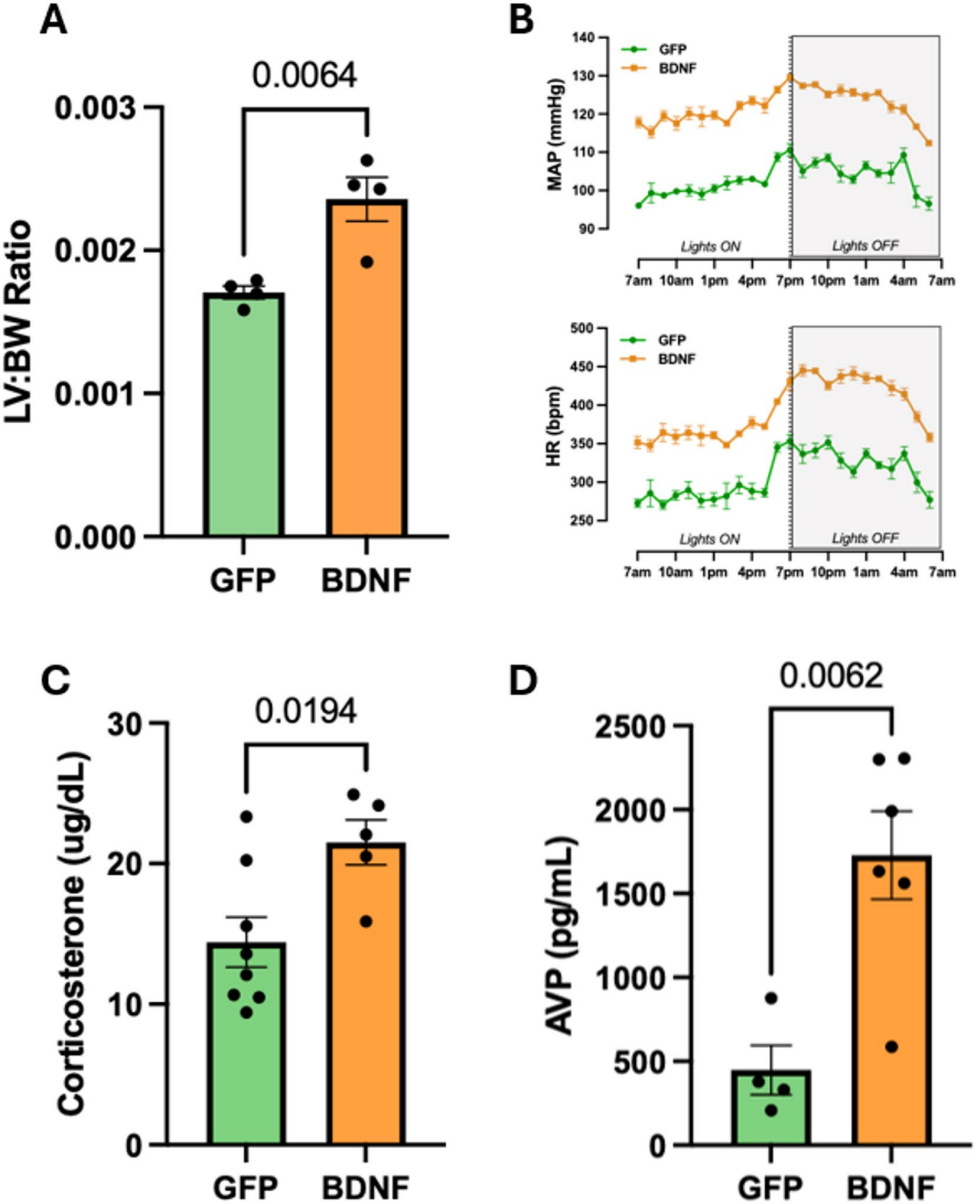
Assessment of BDNF-induced cardiovascular changes and activation of neuroendocrine stress pathways 14 to 19 weeks post viral vector injections. **A**: Cardiac left ventricle weight (LV) to body weight (BW) ratio (left, *n* = 4/group). **B**: Representative radiotelemetric recordings of mean arterial pressure (MAP) and heart rate (HR) averaged over 5 days in a single PVN-BDNF and a single PVN-GFP rat at 14 weeks after vector injections; shaded rectangle indicates nighttime (Lights OFF). **C-D**: Plasma corticosterone (*GFP*: *n* = 8; *BDNF*: *n* = 5) and AVP (*GFP*: *n* = 4; *BDNF*: *n* = 6) levels measured from blood samples collected from isoflurane-anesthetized rats using cardiac punctures before euthanasia. Results are expressed as mean ± SEM. Statistical significance was tested with unpaired t-test.

Measurements in metabolic cages during the in vivo voiding behavior assessment also showed lower daily food intake in PVN-BDNF rats (*Week 14*: GFP: 25.6 ± 1 g/day, BDNF: 21.5 ± 0.8 g/day; *p* < 0.05, two-way ANOVA with Tukey’s test for post hoc analysis), confirming previous evidence^44–46^ of BDNF-mediated actions on food intake and metabolism. In addition, according to previous studies^41,47–50^, we expected to observe activation of key neuroendocrine stress pathways – including the HPA axis, the sympathetic nervous system, and AVP signaling – as well as the development of hypertension in PVN-BDNF rats. Consistent with these expectations, BDNF overexpression led to a significant cardiac left ventricle hypertrophy compared to the control group (Fig. 2A), and radiotelemetry measurements revealed elevated mean arterial pressure and heart rate in PVN-BDNF vs. PVN-GFP rats at 14 weeks after vector injections (Fig. 2B). In addition, plasma levels of corticosterone (Fig. 2C) and AVP (Fig. 2D), were significantly elevated in PVN-BDNF rats compared to controls.

## Effects of the PVN-BDNF model on rat urine voiding behavior

**Fig. 3:**
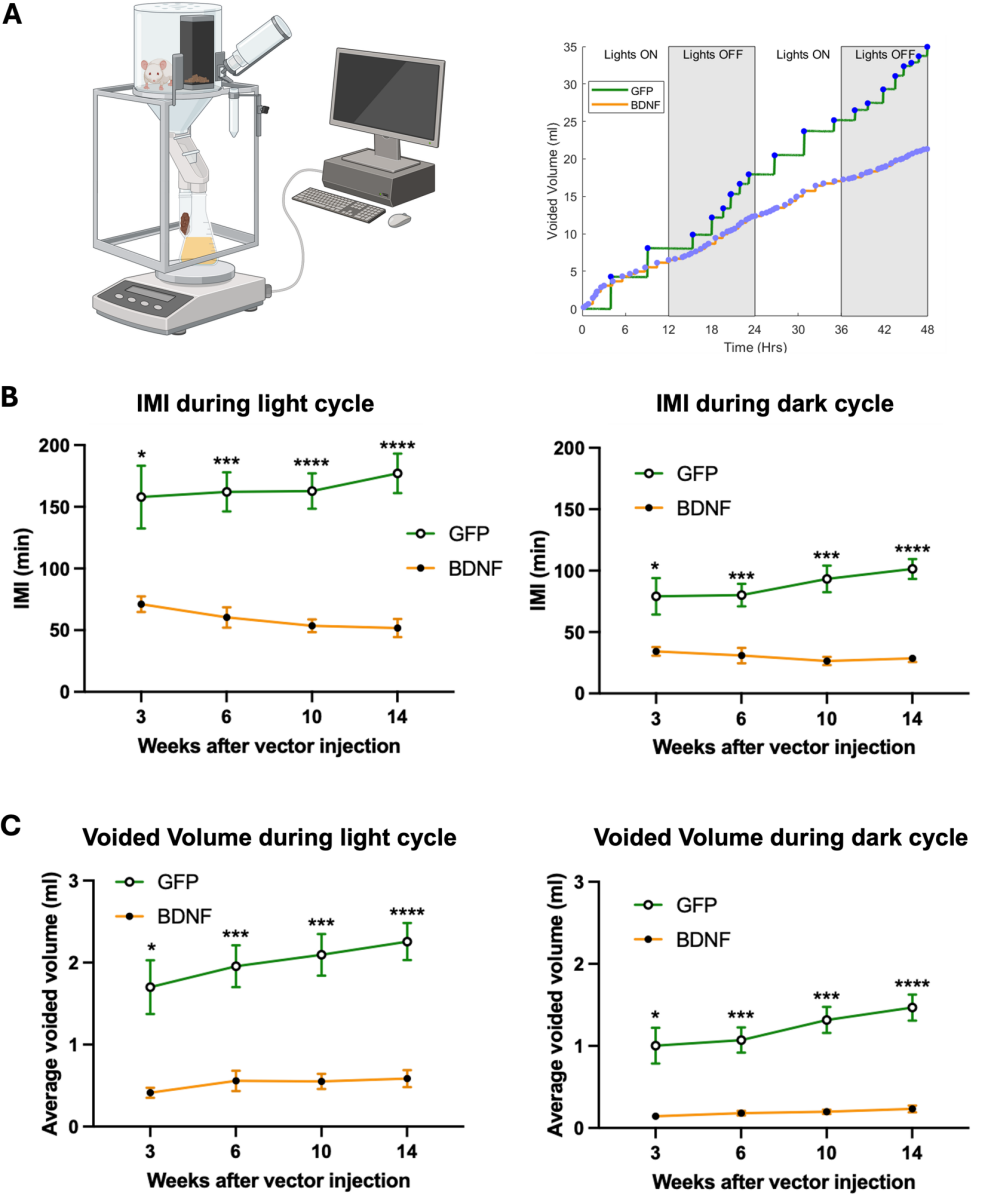
Noninvasive assessment of voiding behavior in PVN-GFP and PVN-BDNF rats at weeks 3, 6, 10, and 14 following vector injections. **A**: UroVoid metabolic cage setup used for urine voiding behavior assessment (left), and representative traces of voiding micturogram at the week 14 timepoint from a PVN-BDNF vs. a PVN-GFP rat (right). **B**: Intermicturition interval (IMI) at all timepoints during the light (left) and dark (right) phases averaged over the 48-hr sessions in PVN-GFP and PVN-BDNF rats. **C**: Voided volume at all timepoints during the light (left) and dark (right) phases averaged over the 48-hr sessions in PVN-GFP and PVN-BDNF rats. Results are expressed as mean ± SEM. Two-way repeated measures ANOVA with Tukey’s post hoc test was used. *p < 0.05, **p < 0.01, ***p < 0.001, ****p < 0.0001 vs. control (GFP: *n* = 8; BDNF: *n* = 6).

Changes in urine voiding behavior patterns in response to chronic PVN-BDNF-induced activation of stress mechanisms were studied in UroVoid metabolic cages at weeks 3, 6, 10, and 14 after viral vector injections (Fig. 3A). We found that BDNF overexpression led to a significant increase in urinary voiding frequency and reduced voided volumes at all time points studied. On average, intermicturition interval (IMI, Fig. 3B) was 2.2-fold higher in GFP vs. BDNF at week 3 (GFP: 101.2 ± 16.5 min, BDNF: 45.1 ± 3.5 min) and became 3.5-fold higher by week 14 (GFP: 123.7 ± 7.7 min, BDNF: 35.7 ± 3.3 min) both during day and nighttime (Group: *p* < 0.0001, Time: *p* = 0.27; Group x Time: *p* < 0.01, mixed-effects model with Tukey’s test for post hoc analysis). On the other hand, differences in voided volume showed a marked circadian fluctuation with PVN-GFP rats having on average ~ 6.5-fold higher voided volume during nighttime (*Week 3*: GFP: 1.0 ± 0.2 ml, BDNF: 0.1 ± 0.02 ml; *Week 14*: GFP: 1.5 ± 0.2 ml, BDNF: 0.2 ± 0.04 ml; Group: *p* < 0.0001, Time: *p* < 0.05, Group x Time: *p* < 0.01) but only ~ 3.8-fold higher voided volume during daytime (*Week 3*: GFP: 1.7 ± 0.3 ml, BDNF: 0.4 ± 0.1 ml; *Week 14*: GFP: 2.3 ± 0.2 ml, BDNF: 0.6 ± 0.1 ml; Group: *p* < 0.001, Time: *p* = 0.48, Group x Time: *p* = 0.10, mixed-effects model with Tukey’s test for post hoc analysis).

**Fig. 4:**
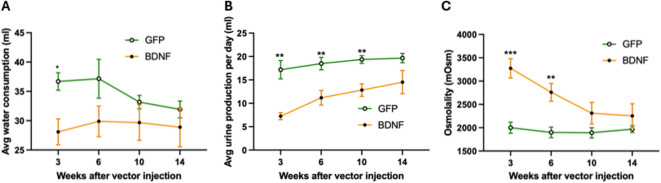
Average water consumption, urine production, and urine osmolality at weeks 3, 6, 10, and 14 following vector injections. **A**: Average daily water consumption observed at all timepoints averaged over the 48-hr sessions in PVN-GFP and PVN-BDNF rats. **B**: Daily urine production at all timepoints averaged over the 48-hr sessions in PVN-GFP and PVN-BDNF rats. **C**: Urine osmolality at all timepoints assessed in urine collected at the conclusion of the 48-hr recording sessions in PVN-GFP and PVN-BDNF rats. Results are expressed as mean ± SEM. A Two-way repeated measures ANOVA with Tukey’s post hoc test was used. *p < 0.05, **p < 0.01 vs. control (GFP: *n* = 8; BDNF: *n* = 6).

BDNF overexpression also modulated water intake, urine production and urine osmolality, with each parameter showing a distinct temporal pattern. Water intake was significantly reduced in PVN-BDNF rats compared to controls at week 3 after vector injections; however, this difference diminished at later timepoints (Group: *p* = 0.08, Time: *p* = 0.17, Group x Time: *p* = 0.12, two-way ANOVA with Tukey’s test for post hoc analysis; Fig. 4A). The average urine production rate was also reduced in PVN-BDNF rats at week 3 compared to control rats, but while the difference diminished similarly to water intake during the experiment, it remained statistically significant until week 10 following vector injections (Group: *p* < 0.001, Time: *p* < 0.01, Group x Time: *p* = 0.12, two-way ANOVA with Tukey’s test for post hoc analysis; Fig. 4B). Comparing urine production rate and voiding behavior, it is important to note that the PVN-BDNF rats demonstrated increased void frequency despite having a significantly lower daily urine production rate for most of the study. In addition, urine osmolality was also markedly elevated in the PVN-BDNF group at weeks 3 and 6 but normalized during the second half of the experiment (Group: *p* < 0.01, Time: *p* < 0.0001, Group x Time: *p* < 0.0001, two-way ANOVA with Tukey’s test for post hoc analysis; Fig. 4C).

## Effects of BDNF overexpression in the PVN on urinary bladder contractility

**Fig. 5:**
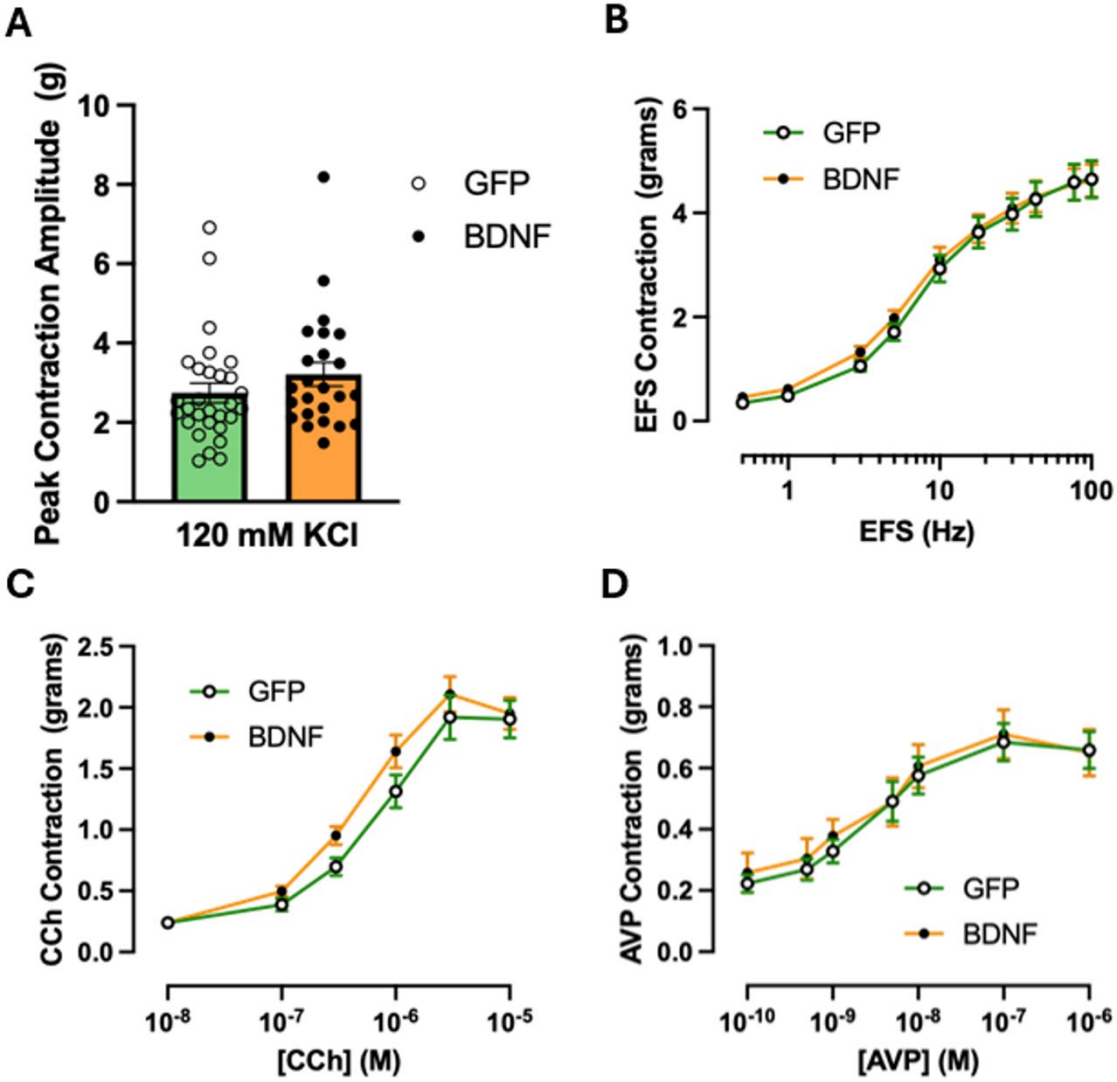
Bladder strip contractility in PVN-GFP and PVN-BDNF groups at weeks 14 to 17 after vector injections. **A**: Peak contraction amplitude in response to 120 mM KCl (GFP: *n =* 30 strips from 8 rats; BDNF: *n =* 24 strips from 6 rats). **B**: Electric Field Stimulation (EFS)-induced bladder strip contractions (GFP: *n =* 30 strips from 8 rats; BDNF: *n =* 24 strips from 6 rats). **C**: Carbachol (CCh)-induced bladder strip contractions (GFP: *n =* 30 strips from 8 rats; BDNF: *n =* 24 strips from 6 rats). **D**: Arginine vasopressin (AVP)-induced bladder strip contractions (GFP: *n =* 26 strips from 7 rats; BDNF: *n =* 20 strips from 5 rats). Unpaired t-test was used for KCl. Two-way repeated measures ANOVA with Tukey’s post hoc test was utilized for EFS and CCh, and a mixed-effects model with Tukey’s post hoc test for AVP. There were no significant group differences in any of the experiments. Results are expressed as mean ± SEM.

Since central activation of the neuroendocrine stress pathways led to altered urinary voiding behavior, we examined whether urinary bladder contractile mechanisms were also impacted. To assess maximal contractile capability of the tissue, bladder strips were subjected to applications of 120 mM KCl, which depolarizes urinary bladder smooth muscle and initiates contraction via Ca^2+^ influx through voltage-dependent calcium channels^53^. Bladder strips from PVN-BDNF and PVN-GFP rats responded similarly to KCl applications (*p* = 0.23; unpaired t-test), suggesting that overall contractile mechanisms are not disrupted by PVN-BDNF treatment (Fig. 5A). Electrical field stimulation (EFS) assessed the nerve-mediated contractions in bladder strips^53^, and administration of increasing concentrations of carbachol (CCh) specifically tested the muscarinic receptor mediated contractions. Overall, BDNF overexpression in the PVN failed to change urinary bladder contractility in response to either EFS (Group: *p* = 0.71) or CCh (Group: *p* = 0.23; two-way ANOVA with Tukey’s test for post hoc analysis), suggesting the local cellular mechanisms impacting contractility were not affected by the PVN-BDNF treatment (Figs. 5B-C). Lastly, AVP receptor-mediated contractions were compared between the two groups to examine whether those were affected by chronically elevated plasma AVP levels in PVN-BDNF rats. However, AVP-induced contractions were also found to be similar in PVN-BDNF and PVN-GFP groups (Group: *p* = 0.67; mixed-effects model with Tukey’s test for post hoc analysis; Fig. 5D).

## Effects of BDNF overexpression in the PVN on bladder wall mechanics and bladder capacity

**Fig. 6:**
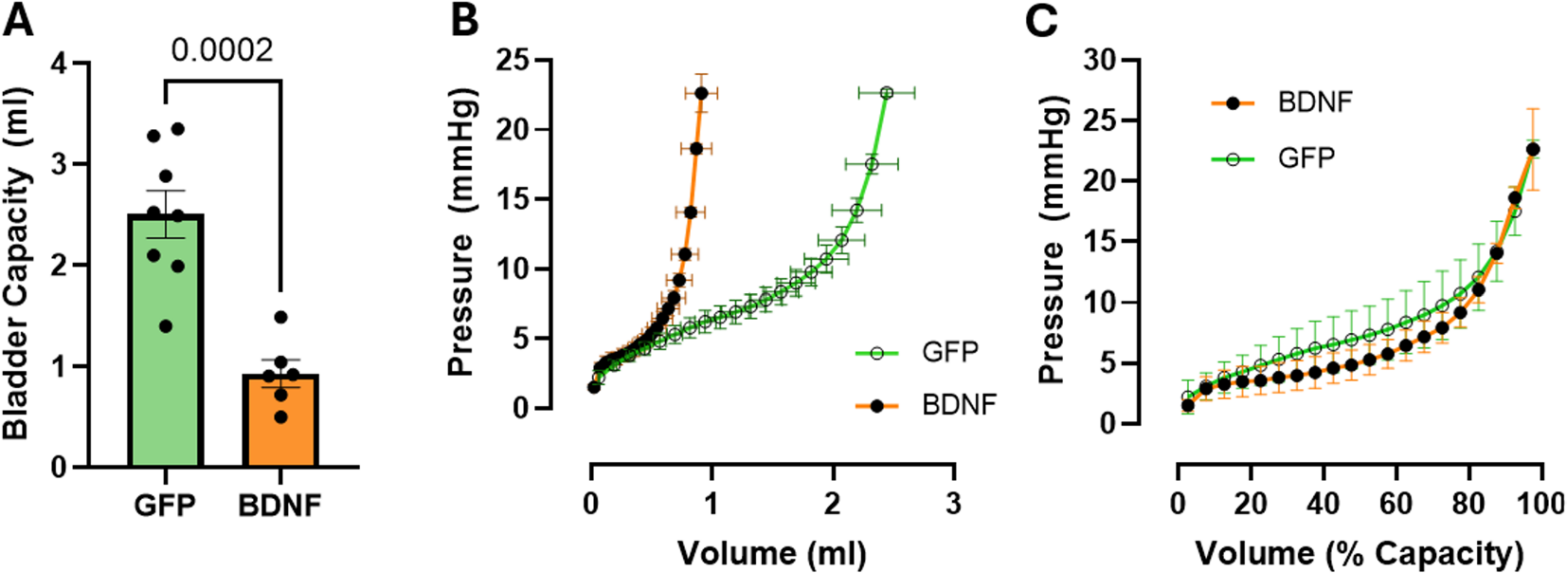
Bladder capacity and volume-pressure curves in ex vivo pressurized bladders from PVN-GFP and PVN-BDNF rats at weeks 17 to 19 after vector injections. **A**: Bladder capacity, defined as the volume a urinary bladder holds once the pressure reaches 25 mmHg. **B**: Bladder pressure vs. infused volume. **C**: Bladder pressure vs. infused volume expressed as % of bladder capacity). Unpaired t-test was used for bladder capacity and two-way repeated measures ANOVA with Tukey’s post hoc test was used for volume-pressure curves (GFP: *n* = 8; BDNF: *n* = 6). Results are expressed as mean ± SEM. *P values are shown above data sets.*

Despite the apparently normal detrusor contractility in urinary bladder strips isolated from PVN-BDNF rats, changes in bladder wall mechanics could still impact voiding behavior in vivo. To investigate this possibility, we measured pressure-volume relationships in isolated pressurized urinary bladders ex vivo. Bladders were filled with physiological saline solution at a constant rate until bladder pressure reached 25 mmHg. We defined the volume of the bladder at 25 mmHg as the maximal bladder capacity^53–55^. We found that maximal capacity measured ex vivo in bladders from PVN-BDNF rats was significantly lower than maximal capacity measured in bladders from PVN-GFP controls (*GFP*: 2.50 ± 0.23 ml, *BDNF*: 0.93 ± 0.13 ml, *p* < 0.001, unpaired t-test; Fig. 6A). This difference is also reflected in the markedly steeper rise of bladder pressure in the PVN-BDNF group during bladder filling compared to PVN-GFP (Group: *p* < 0.0001, Bladder pressure: *p* < 0.0001, Group x Bladder pressure: *p* < 0.0001; two-way ANOVA with Tukey’s test for post hoc analysis; Fig. 6B). However, the relationship between bladder pressure and normalized bladder capacity was similar between the two groups (Group: *p* = 0.14; two-way ANOVA with Tukey’s test for post hoc analysis; Fig. 6C) indicating that PVN-BDNF treatment primarily affected bladder volume and not elasticity of the bladder wall. In other words, bladders from PVN-BDNF rats appear physically smaller and hold less volume than bladders from PVN-GFP control rats.

## Bladder wall morphology is significantly impacted by the PVN-BDNF model

**Fig. 7:**
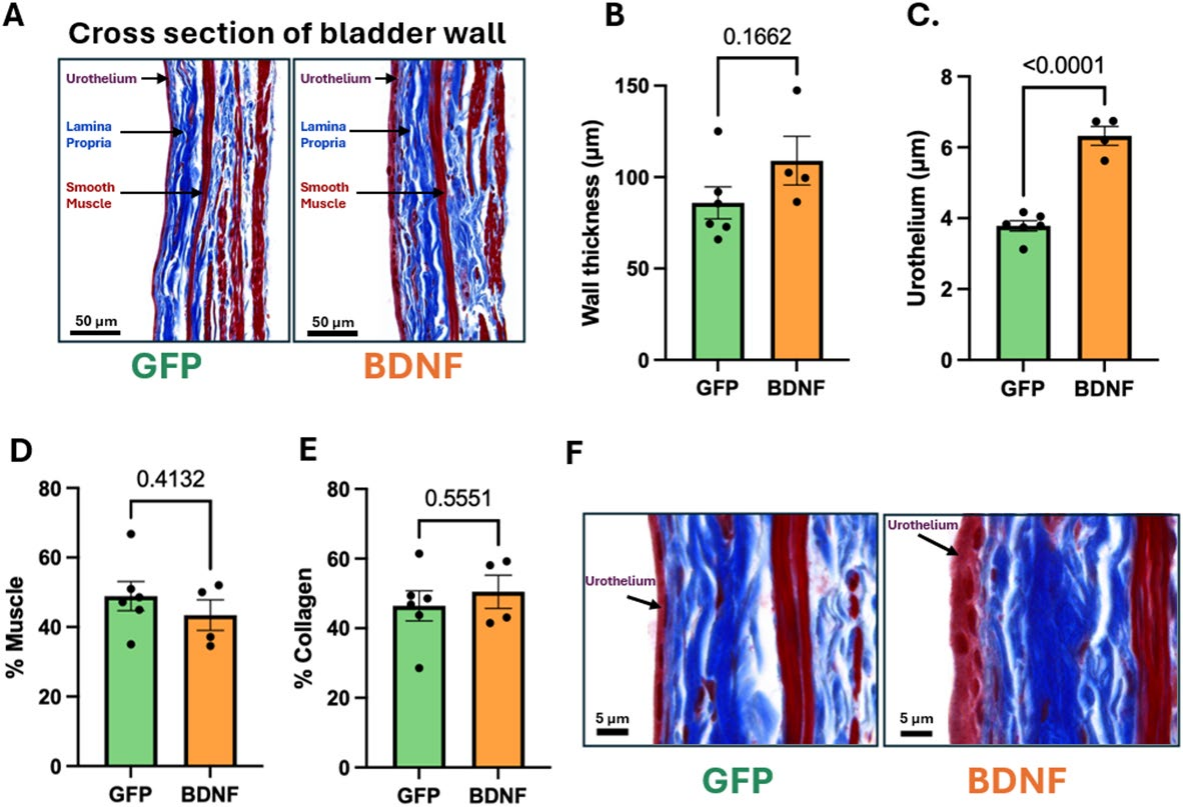
Assessment of urinary bladder wall morphology in PVN-GFP and PVN-BDNF bladders fixed at 25mmHg pressure at weeks 17 to 19 after vector injections. **A**: Representative images of PVN-GFP vs. PVN-BDNF Masson’s trichrome-stained sections of the bladder wall at 40x magnification (urothelium: dark red/purple cellular layer on the left; collagen: blue; smooth muscle cells: red). **B**: Overall bladder wall thickness. **C**: Thickness of the urothelium. **D-E**: Percent smooth muscle and collagen content. **F**: Representative images of a PVN-GFP and a PVN-BDNF bladder sections taken at 63x magnification showing morphological changes in the urothelium. Results are expressed as mean ± SEM. An unpaired t-test was used. *P values are given above the group comparisons. (*GFP: *n* = 6; BDNF: *n* = 4*).*

Urinary bladder wall histology was performed in ex vivo bladders that were filled to 100% capacity at 25 mmHg pressure before being fixed in 4% paraformaldehyde (PFA). Overall bladder wall thickness was similar between the two groups, and proportions of smooth muscle and collagen content were also unaffected by the PVN-BDNF treatment (Fig. 7A-B, D-E). In contrast, the urothelium was significantly thicker and cells also appeared to be more rounded in bladders from the PVN-BDNF group compared to PVN-GFP (Fig. 7C, F). In addition, overall bladder weights were similar in the two groups (*GFP*: 0.18 ± 0.03 g; *BDNF*: 0.13 ± 0.02 g; unpaired t-test) despite the significantly reduced capacity of the PVN-BDNF bladders compared with PVN-GFP. Thus, bladders from PVN-BDNF rats demonstrated a significantly higher weight per surface area (0.77 ± 0.1 µg/mm^2^) compared to PVN-GFP (0.35 ± 0.05 µg/mm^2^; *p* < 0.05; unpaired t-test).

## Discussion

OAB is a chronic medical condition, and psychological stress or stress-associated disorders are important contributing factors to the development of this condition^5–10^. However, the role of neuroendocrine stress mechanisms relative to psychological or environmental factors remain to be characterized in this bladder pathology. Therefore, based on previous studies of BDNF-mediated mechanisms in the PVN^41,47–50^, we developed a novel model of prolonged BDNF overexpression to activate neuroendocrine stress pathways controlled in the PVN. This approach offered two key advantages. First, it enabled the examination of chronic activation of neuroendocrine stress pathways on bladder function and the central control of micturition over a much longer period than commonly used in chronic stress protocols. Second, it allowed us to isolate the adverse effects of activated neuroendocrine stress mechanisms from variability arising due to behavioral aspects of stress paradigms. Confirming our model-induced PVN-BDNF phenotype, all animals with successful bilateral BDNF viral vector injections showed lower body weight gain, elevated blood pressure, cardiac left ventricle hypertrophy and higher corticosterone and AVP plasma levels, indicating successful activation of neuroendocrine stress mechanisms along with significant metabolic and cardiovascular changes without subjecting rats to environmental or psychological stressors.

Noninvasive voiding behavior assessment revealed that rats receiving PVN-BDNF treatment showed significantly disrupted voiding behavior compared to control animals indicating that activation of neuroendocrine stress mechanisms without the behavioral or environmental aspects of external stressors are sufficient to induce lower urinary tract symptoms. PVN-BDNF rats had more frequent, smaller volume voids than PVN-GFP control rats, consistent with an OAB phenotype. These changes were observed during both day- and nighttime within three weeks of vector injections and the differences remained for the whole duration (14 weeks) of the study. Previous research on bladder overactivity has suggested that an OAB phenotype could eventually lead to underactivity caused by bladder denervation^56^. However, we did not observe such changes in the PVN-BDNF model despite the considerable length of observation, equivalent to about a decade in human years^57^. This suggests that either longer time is needed for overactivity to transition into underactivity or that interactions of specific behavioral or psychological factors are necessary for such change in voiding behavior.

Central activation of neuroendocrine stress mechanisms modulated daily water consumption, urine production and urine osmolality. Notably, water intake was reduced initially in PVN-BDNF rats and was significantly lower compared with controls at the week 3 timepoint. However, this difference between the two groups diminished in later stages of the study. Parallel with lower water intake, urine production also decreased initially in the PVN-BDNF group, remaining significantly reduced until the week 10 timepoint, and urine osmolality stayed elevated up to 6 weeks post vector injections indicating that chronic activation of neuroendocrine stress mechanisms, in particular AVP signaling^58,59^, significantly influenced water/electrolyte homeostasis in our model. However, all these effects diminished over time. This pattern indicates that changes in water intake, urine production and osmolality are unlikely to be caused by a difference in body weight, which became progressively greater during the study. Interestingly, while urine production was reduced in PVN-BDNF rats, their voiding frequency was still significantly higher than that of control rats during these early stages of the experiment, and this group difference in voiding behavior persisted throughout the experiment even as differences in urine production diminished. It is also important to note that the reduction in voided volume in PVN-BDNF rats is far greater than would be expected based on their lower body weight and voided volume remains significantly different between groups even after normalization to body weight (Supplementary Fig. 1). Besides the well-described renal effects of AVP promoting water reabsorption and reducing urine production, AVP can also exert direct effects on the bladder either via blood circulation or by its clearance into urine^58,60^ and our data shows that circulating AVP levels remain high for the duration of the experiment. Thus, the OAB phenotype seems to be independent of AVP’s actions on renal function and urine production. However, AVP could still exert direct effects on the bladder triggering adaptive changes in voiding frequency and voided volume that persist despite the normalization of urine production later.

The impact of stress on circadian patterns in voiding behavior is highly understudied in animal research. Many behavioral stress exposure paradigms, such as chronic or repeated variate stress, restraint stress, water avoidance stress, and social defeat stress, are administered during the daytime^13–16,18,19,26,30,32,33,61–67^. Moreover, most of these studies either only look at the overall voiding behavior differences (not delineating between daytime and nighttime patterns) or separate the daytime and nighttime voiding behaviors but still administer the stressors during the daytime. This influences any circadian variations in voiding behavior observed in those studies, unsurprisingly finding the main differences during daytime when the animals are being subjected to stressors^32^. Alternatively, the PVN-BDNF model provides a continuous central activation of neuroendocrine stress pathways, affecting animals during both their active *and* inactive periods possibly better imitating continuous stress exposure. Notably, we observed a significant circadian effect: compared to controls, PVN-BDNF rats exhibited a markedly greater reduction in voided volume during their active (nighttime) vs. inactive (daytime) period. Cortisol (in humans) and corticosterone (in rodents) both have circadian rhythms that modulate and optimize metabolic responses^68^ and can affect factors such as nocturia^69^, major bladder clock genes, bladder peripheral clock and diurnal micturition patterns^70^. However, in our PVN-BDNF model, increased HPA axis activity is driven by viral vector-mediated BDNF expression, which is less likely to be affected by circadian rhythms. Therefore, it seems that there may be additional circadian mechanisms, either in the central nervous system or in the bladder, that control voided volume and are amplified by the PVN-BDNF treatment-induced neuroendocrine stress mechanisms. One limitation of our noninvasive voiding-behavior assessment is the need for single housing in a metabolic cage, which introduces additional stress. Although PVN-GFP and PVN-BDNF rats were subject to this stressor equally, and the animals were acclimated to the metabolic cage for ~ 17 hr before recordings began to minimize this effect, there is still a possibility that BDNF upregulation in the PVN could modulate the response and influence voiding behavior by augmenting the effect of single housing.

Previous research has shown that stress-induced bladder overactivity is associated with enhanced urinary bladder contractility in some models^18,63,71^; however, the underlying mechanisms are not yet well characterized. A potential link between enhanced contractility and OAB is that the detrusor smooth muscle exhibits non-voiding contractions during bladder filling, and these transient contractions are correlated with increased afferent nerve activity^54^. Therefore, increased bladder contractility could in theory drive higher afferent nerve activity leading to bladder dysfunction. However, our results indicate that enhanced contractile mechanisms in the detrusor muscle is not a major underlying mechanism of OAB in the PVN-BDNF model. We found no differences in maximum contractile force in response to high K^+^ concentration-induced depolarization between the PVN-BDNF and control groups. A dual approach of using EFS to assess nerve-mediated contractions^53^ and CCh to specifically test muscarinic receptor mediated contractions found no significant group differences either. In rats, release of adenosine triphosphate (ATP) from parasympathetic terminals contributes significantly to detrusor contraction^72^. Although we did not directly assess purinergic receptor-mediated contractility, the fact that EFS activates parasympathetic nerves and thereby releases ATP, and that EFS-evoked contractions were similar in bladder strips from GFP and BDNF rats, indicate that the PVN-BDNF model is unlikely to produce substantial changes in this mechanism either. Thus, our results suggest that changes in smooth muscle contractility and EFS-induced neurotransmitter release are not expected to drive the OAB phenotype induced by chronic activation of neuroendocrine mechanisms in our model. Since PVN-BNDF treatment leads to elevated plasma AVP, and AVP receptors are expressed both in the bladder mucosa and detrusor muscle^60^, AVP-evoked contractions were also assessed but were found to be similar in the two groups. While these findings indicate that responsiveness of the bladder to AVP did not change, they also raise the possibility that high circulating AVP levels in our model, and potentially also during chronic stress^41^, could lead to increased bladder wall contractility and OAB in vivo due to the lack of compensatory downregulation of AVP-mediated bladder contractions. In this study, we did not examine urodynamic properties of bladder function using cystometry. It is possible that PVN-BDNF rats manifest increased non-voiding contractions in vivo, and this could be a mechanism that would drive increased afferent nerve output from the bladder during filling^54^. However, in the present study, we did not observe any striking differences in transient phasic contractions in isolated bladder strips during contractile studies or in transient pressure events in ex vivo pressurized bladder studies; thus, we do not anticipate that changes in non-voiding contractions would underlie the apparent OAB phenotype we observe in PVN-BDNF rats. However, this possibility needs to be specifically addressed in cystometry studies in future work.

Reduced bladder capacity could be another explanation for overactivity observed in our model. The lower voided volume reported in PVN-BDNF rats during in vivo voiding behavior assessments does not provide any information about how much volume a bladder can maximally hold. It is possible that bladders from PVN-BDNF rats have the capacity to hold the same volume as bladders from PVN-GFP rats, but central regulation triggers micturition before the bladder is physically full. However, pressurized ex vivo bladder experiments indicated that in fact, bladders from PVN-BDNF rats had lower capacity than bladders from PVN-GFP rats. These bladders also showed steeper volume-pressure relationships, with bladder pressure rising more for a given change in volume than in bladders from PVN-GFP rats. To explore if this change in bladder volume-pressure relationship is due to altered bladder wall mechanics, we also expressed bladder pressure as a function of volume normalized to bladder capacity. This relationship was largely similar in bladders from PVN-BDNF and PVN-GFP rats. Thus, bladder wall mechanics and elasticity are not likely to contribute to the altered volume-pressure curve. Instead, it seems that bladders from PVN-BDNF rats are just physically smaller than bladders from PVN-GFP rats. However, an interesting question is whether reduced bladder capacity develops first, potentially during the transient phase of reduced urine production, and becomes a major driver of overactivity later, or whether reduced bladder capacity is the consequence of the centrally driven increase in voiding frequency leading to bladder wall remodeling to match the reduced voided volume.

Previous findings in rodents have shown that stress can lead to urinary bladder wall remodeling^11,12,16,17,61,73^. In our experiments, we found that overall bladder weights were similar in the PVN-BDNF and PVN-GFP groups despite the significantly smaller capacity of bladders from PVN-BDNF rats, which resulted in markedly higher weight per surface area in the PVN-BDNF group compared to PVN-GFP controls. Urinary bladder wall histology also showed no statistically detectable difference in overall wall thickness between the two groups at our current sample size, despite the significantly smaller size of bladders from PVN-BDNF rats indicating the development of relative bladder wall hypertrophy in the PVN-BDNF model. However, proportions of smooth muscle and collagen content remained unaffected by the PVN-BDNF treatment in agreement with no difference in the normalized volume-pressure curves. In contrast, the urothelium was significantly thicker in bladders from PVN-BDNF rats compared to PVN-GFP controls with some morphological changes also noted in urothelial cells of PVN-BDNF rats compared to PVN-GFP controls. While it’s unclear whether the change in urothelium thickness is biologically meaningful, it may reflect important underlying mechanisms activated by the PVN-BDNF model potentially impacting urothelium-dependent barrier, sensory and contractile mechanisms^74–78^. Such mechanisms may involve upregulation of trophic factors, including nerve growth factor, which has been implicated in stress-related bladder dysfunction^34,67^ and shown to induce bladder hypertrophy^79^, as well as circulating AVP, given its marked elevation in PVN-BDNF rats, the presence of both V_1a_ and V_2_ receptors throughout the bladder wall, and its established hypertrophic effects in vascular, cardiac, and renal tissues^80–82^.

In summary, this study aimed to investigate the pathophysiological mechanisms of chronic neuroendocrine stress-induced OAB without any variability introduced by environmental or behavioral aspects of chronic stress paradigms. To do this, we used a model of vector-mediated BDNF overexpression within the PVN to centrally activate the different arms of the stress response for a long duration so we could investigate their impact on bladder function. We demonstrated that activation of neuroendocrine stress mechanisms without environmental or psychological stressors is sufficient to induce a significant OAB phenotype characterized by circadian variation, reduced bladder capacity, relative bladder wall hypertrophy, and morphological changes in the urothelium with no impact on local signaling pathways mediating bladder contractility. Future research will need to focus on underlying mechanisms that contribute to OAB by exploring: (1) the urothelium and afferent signaling, (2) mechanisms affecting bladder capacity, (3) the central circuitry, especially connections between PVN neurons and the micturition center, and (4) the impact of hypertension and cardiovascular dysfunction. A significant limitation of this study is that it only included male rats, thus not exploring sex-dependent phenotypic differences in voiding behavior and bladder function. An important reason for using only male rats for the current study was that our previous findings indicated that the PVN-BDNF treatment led to significantly enhanced phenotype in male rats with higher blood pressure, heart rate, and corticosterone levels and more significant metabolic effects compared to females^48^. Thus, direct comparison of males and females would not be possible using our model. Altogether, the PVN-BDNF model provides a unique framework for future studies identifying pathways that contribute to bladder dysfunction during prolonged activation of neuroendocrine stress mechanisms and associated cardiovascular changes and notably represents the first demonstration of an OAB-like phenotype occurring without environmental or external behavioral stressors.

## Materials and methods

### Animal use

Seventy-eight male Sprague Dawley rats were purchased from Charles River, Canada at the age of 9 weeks, housed in pairs at the University of Vermont Animal Care Facility. Rats were acclimated to the location for a minimum of five days before handling and maintained on a 12-hour light/dark cycle with access to food and water ad libitum. Rats were randomly assigned into control and experimental groups for viral vector injections and were weighed daily during the 1-week post-operative monitoring and bi-weekly after that. All experimental protocols and procedures were approved by the University of Vermont’s Institutional Animal Care and Use Committee and conducted according to the National Institutes of Health (NIH) Guide for the Care and Use of Laboratory Animals. The study procedures are reported according to the ARRIVE guidelines (Animal Research: Reporting of In Vivo Experiments).

### Experimental design

The first experiment tested the hypothesis that PVN-BDNF treatment would lead to changes in voiding behavior, water intake and urine osmolality. Rats were taken from their home cages and placed in UroVoid metabolic cages to assess each of these parameters at weeks 3, 6, 10, and 14 post injections. Additional experiments tested the hypothesis that PVN-BDNF treatment would alter bladder contractility mechanisms, bladder wall mechanics, bladder capacity, and bladder morphology. These experiments were conducted on bladders freshly isolated from animals euthanized 14 to 19 weeks following viral vector injections.

### Surgical procedures and euthanasia

Surgeries were completed using aseptic techniques under isoflurane anesthesia (5% induction, 2–3% maintenance in oxygen). The animal’s reflex response to a toe pinch on the hind paw was employed to gauge the depth of anesthesia before any surgical procedures were performed. Body temperature, heart rate and blood oxygenation were monitored, and a heating pad was controlled by a Kent Scientific PhysioSuite system. Carprofen was administered for post-operative analgesia at the start of surgery (5 mg/kg/day s.c.) and for two days following the surgery (2 mg tablet/day).

#### Viral vector-mediated gene expression in the PVN

Adeno-associated virus-2 (AAV2) viral vectors were produced and packaged by Vector Biolabs (Philadelphia, PA, USA) to express either a rat BDNF-myc tag construct (BDNFmyc) or green fluorescent protein (GFP). The chicken-β-actin promoter with human cytomegalovirus enhancer and a woodchuck post-transcriptional regulatory element drove and enhanced the expression of GFP and BDNFmyc. The BDNFmyc plasmid, a generous gift from Dr. Ronald Klein (LSU Health Sciences Center Shreveport, LA, USA), has already been utilized in protection of retinal ganglion cells in a rat glaucoma model^83^ and in assessment of BDNF-mediated cardiovascular effects in the PVN^47–50,84^.

At 10–11 weeks of age, rats were anesthetized using isoflurane and transferred to a stereotaxic surgery frame where they received bilateral PVN injections of AAV2 viral vectors (10^12^ viral particles/mL; 200 nL/side) using glass micropipettes derived from thin-walled borosilicate glass capillary tubes (World Precision Instruments Inc., Sarasota, FL, USA) pulled to the following dimensions: OD, 1 mm; ID, 0.58 mm; tip diameter, ~ 25 μm. The injections were performed with the micropipettes angled 10º laterally toward the midline at stereotactic coordinates located 1.80 mm posterior to bregma, 1.70 mm lateral to the midline, and 7.65 mm ventral from the dorsal surface of the brain. Vector solutions were injected using a pneumatic pico pump (World Precision Instruments, Sarasota, FL) over a 5 min period with the micropipette being left at the site of injection for 3 more min before being withdrawn.

#### Radiotelemetric transmitter implantation

Ten weeks after the viral vector injections, a subgroup of rats had a radiotelemetric transducer (model HD-S10; Data Sciences International, St. Paul, MN, USA) implanted into the descending aorta through a midline abdominal incision as previously described^48,49^. Dataquest A.R.T. Analysis software was used to analyze the animals’ blood pressure and heart rate from data recordings captured every 10 min for 15 s and averaged for 5 days during week 14 following vector injections.

#### Euthanasia and transcardial perfusion

At 14–19 weeks after viral vector injections, rats were put under 5% isoflurane anesthesia. An incision was first made through the abdominal walls, below the ribcage and then through the ribcage to the collarbone to expose the heart. Rats were either transcardially perfused with 400 mL of ice cold 1x phosphate buffer solution (PBS) or with 400 mL of ice cold 1x PBS followed by 400 mL of ice-cold 4% PFA. Tissue samples were collected as specified in subsections below.

### Tissue harvesting and assay protocols

#### Cardiac left ventricle hypertrophy

Following perfusion with ice cold 1x PBS, the heart was immediately extracted, and the left ventricle was isolated and weighed using a high precision weighing scale (Mettler Toledo, USA). Each left ventricle weight was then normalized to the animal’s body weight.

#### Measurement of plasma AVP and corticosterone levels

Blood samples were obtained under isoflurane anesthesia from cardiac punctures with a 3 ml syringe attached to an 18-gauge needle before transcardial perfusion. Blood samples were collected in a 2 mL vacutainer tube containing sodium citrate and centrifuged at 4 °C for 10 min at 2600 rpm. Plasma was then extracted and stored at −80 °C. Plasma AVP and CORT levels were assessed using commercially available enzyme-linked immunosorbent assay kits (ThermoFisher Scientific, USA, Catalog # EIAAVP & Enzo Life Sciences, USA, Catalog # ADI-900-097, respectively) as per the manufacturers’ instructions.

#### Immunofluorescence assays and verification of viral vector injections

Following perfusion with 4% PFA, brains were post-fixed in 4% PFA for 2 h before being transferred to 30% sucrose solution to equilibrate at 4 °C. After the brains sank to the base of the container, 40 μm coronal slices were obtained using a freezing stage, sliding microtome (Leica SM2000R). Brain slices were mounted on Fisher Superfrost Plus slides. Immunofluorescence was performed to detect BDNFmyc expression in the PVN using a mouse anti-c-Myc primary antibody (Santa Cruz-9E10, 1:200, overnight incubation at 4 °C) paired with a donkey anti-mouse 488 secondary antibody (Abcam, 1:200, 2 h. incubation at room temperature). GFP and BDNFmyc expression within the PVN was confirmed using a Nikon A1R-HD point scanning confocal microscope that was supported by NIH award # 1S10OD025030-01 from the Office of Research Infrastructure Programs at the University of Vermont’s Microscopy Imaging Center (RRID# SCR 018821).

### Noninvasive voiding behavior assessment for 48 h

Spontaneous rat urinary voiding behavior was assessed repeatedly at weeks 3, 6, 10 and 14 post viral vector injections. Rats were individually placed in metabolic cages (Rodent Caging, Ancare Corp., NY, USA; 13.9 cm in interior height, and 20.32 cm in diameter). Urine was collected in an Erlenmeyer flask placed on a scale that was connected to a data-acquisition computer running UroVoid Data Acquisition Software (Med Associates, Inc., Georgia, VT). The number of urinary voids, intermicturition interval (IMI) and urinary voided volumes along with water and food intake were monitored over a 48-hour period at each timepoint. Osmolality was also assessed with the urine collected at the end of each study session. Rats were placed in the metabolic cages the evening before each session began to allow for an acclimation period. The void recordings spanned a 48-hour period, and started at 6 a.m. Rats were kept on a 12:12 h light: dark cycle during the UroVoid study (lights on at 6 AM). Rats had ad lib access to food and water. Water intake was quantified daily during the study. At the conclusion of each experimental session, the animals were placed back in their home cages with their cage mates.

### Bladder contractility assessments

Under deep isoflurane anesthesia, rat bladders were extracted before the onset of transcardial perfusion and placed in the dissection saline (in mM: 80 monosodium glutamate, 55 NaCl, 10 glucose,10 *N*−2-hydroxyethylpiperazine-*N*’−2-ethanesulfonic acid, 6 KCl, 2 MgCl_2_, and NaOH for pH adjustment to 7.3). Freshly isolated bladders were dissected to expose the urothelial surface. The base of the bladder, including the urethral and ureteral orifices, was removed and the bladder wall was cut into four equal-sized strips (2- to 3-mm wide and 5- to 7-mm long). Bladder strips were then mounted in a tissue bath myograph filled with physiological saline solution (PSS; in mM: 119 NaCl, 24 NaHCO_3_, 11 glucose, 4.7 KCl, 1.2 KH_2_PO_4_, 2.5 CaCl_2_, and 1.2 MgSO_4_), aerated with 20% O_2_ and 5% CO_2_, 75% N_2_ and maintained at 37 °C. A custom lab-built 3D printed myograph (10 ml volume organ baths) was used to assess contractility. Contractility was measured using Radnoti force transducers, and data was recorded using LabChart with a sample rate of 100 Hz.

Bladder strips were first stretched to 1 g of tension and left to equilibrate for 45 min to achieve stable level of force before assessment of contractility as described before^85,86^. After this stabilization period, bladder strips were exposed to three applications of potassium chloride (KCl: 120 mM, 5 min duration) followed by five washes with PSS to clear away any remnants of the solution. The KCl response was used to gauge any potential group differences in peak amplitude of contractions generated by the bladder strips. To assess group differences in nerve-mediated contractions, the amplitude of bladder contractions was analyzed in response to electrical field stimulation (EFS: 20 V pulse amplitude, 0.2 ms pulse width, 2 s duration) at stimulus frequencies of 0.5, 1, 3, 10, 18, 30, 43, 55, 77 and 100 Hz using a pair of platinum wire electrodes that sat parallel to the bladder strip in the tissue bath^53^. Polarity was switched pulse-to-pulse to avoid electrode polarization. Muscarinic receptor mediated contractions were studied using increasing concentrations of carbachol (CCh; 0.01–10 μm) in the tissue bath. Similarly, AVP receptor evoked contractions were assessed using increasing concentrations of AVP (AVP; 0.1 nM − 1 μm).

### Pressurized bladder experiments

Bladder capacity measures were conducted in bladders extracted under deep isoflurane anesthesia before transcardial perfusion. Bladders were placed in cold dissection saline. Ureters of freshly isolated bladders were carefully ligated using a 5/0 silk suture before bladders were transferred to a recording chamber filled with PSS maintained at 37 °C and 7.4 pH, aerated with 20% O_2_−5%CO_2_−75% N_2_. A syringe pump (model 4400-001; Harvard Apparatus) and in-line pressure transducer (PT-F; Living Systems Instrumentation, Saint Albans, VT) attached to a signal conditioner (model NL-108; Digitimer, Hertfordshire, UK) set to 100 mV/cmH_2_O output enabled continuous PSS infusion and bladder pressure recording (respectively) via urethral cannulation. Power3A analog-to-digital converter and Spike2 software (Cambridge Electronic Design, Cambridge, UK) were used for data acquisition. Bladders were infused at a rate of 10 mL/hr until a pressure of 25 mmHg was reached, after which they were emptied through the urethral cannula. This was done at least three times, with a 5 min stabilization period between filling cycles, to obtain stable urodynamic profiles. Bladder capacity was defined as the fluid volume in the bladder necessary to achieve a pressure of 25 mmHg. Because bladder capacity usually stabilizes by the third infusion cycle, bladder capacity measurements were taken from the third filling cycle.

### Bladder histology

At the end of the pressurized bladder experiments, bladders were again pressurized to 25 mmHg. Bladders were then lifted from the recording chambers and fixed in 4% PFA for a week. After fixation, bladders were stabilized in agarose to eliminate folds and wrinkles, embedded in paraffin and sectioned into 5 μm slices. Bladder wall thickness and tissue composition were evaluated using Masson’s trichrome staining, which differentiates muscle, collagen, and other connective tissue components. White pixels were excluded from the analysis to account for any gaps in the tissue. Images were captured at the University of Vermont’s Microscopy Imaging Center (RRID# SCR 018821) using the Leica-Aperio VERSA 8 whole slide imager purchased via a College of Medicine Shared Instrumentation Award. A Fiji software macro was developed to perpendicularly scan through the bladder wall in 40x images to evaluate the composition of the bladder wall based on the color of the stained tissue.

### Statistical analyses

Data are presented as means ± SEM. GraphPad Prism 10.4 (GraphPad Software, Boston, MA) was used for data analyses and findings were either analyzed with a two-tailed unpaired t-test if comparing two overall group means, or with a repeated-measures two-way analysis of variance (ANOVA) paired with a post-hoc Tukey’s test for multiple comparisons if comparing the within-subject changes over time or dose. However, in cases of missing data values, a mixed effect model with a post-hoc Tukey’s test for multiple comparisons was utilized instead. A *P* value less than 0.05 was used as a criterion for statistically significant results and rejection of null hypothesis. The number of animals used for our study was determined from previous studies using similar methodology^53,55^.

## Supplementary Information

Below is the link to the electronic supplementary material.

Supplementary Material 1

Supplementary Material 2

## Data Availability

Data can be made available upon reasonable request. Please contact the corresponding author Benedek Erdos at [berdos@uvm.edu](mailto: berdos@uvm.edu) for data requests.
