# Publisher Correction: Overactive bladder phenotype induced by chronic activation of hypothalamic neuroendocrine stress pathways in rats with no extrinsic behavioral stress applied

**DOI:** 10.1038/s41598-026-36865-9

**Published:** 2026-01-23

**Authors:** Jenan Husain, Alexandra Bakhareva, Anna Pace, Maria Noterman-Soulinthavong, Gerald M. Herrera, Benedek Erdos

**Affiliations:** Department of Pharmacology, University of Vermont, Burlington, 05401 USA

Correction to: *Scientific Reports* 10.1038/s41598-025-32428-6, published online 21 December 2025

The original version of the Article contained errors in Figure 1, 2, 4 and 6. Figure 1 was published as Figure 6.

The original Fig. 1 and the accompanying legend appear below.

**Fig. 1:**
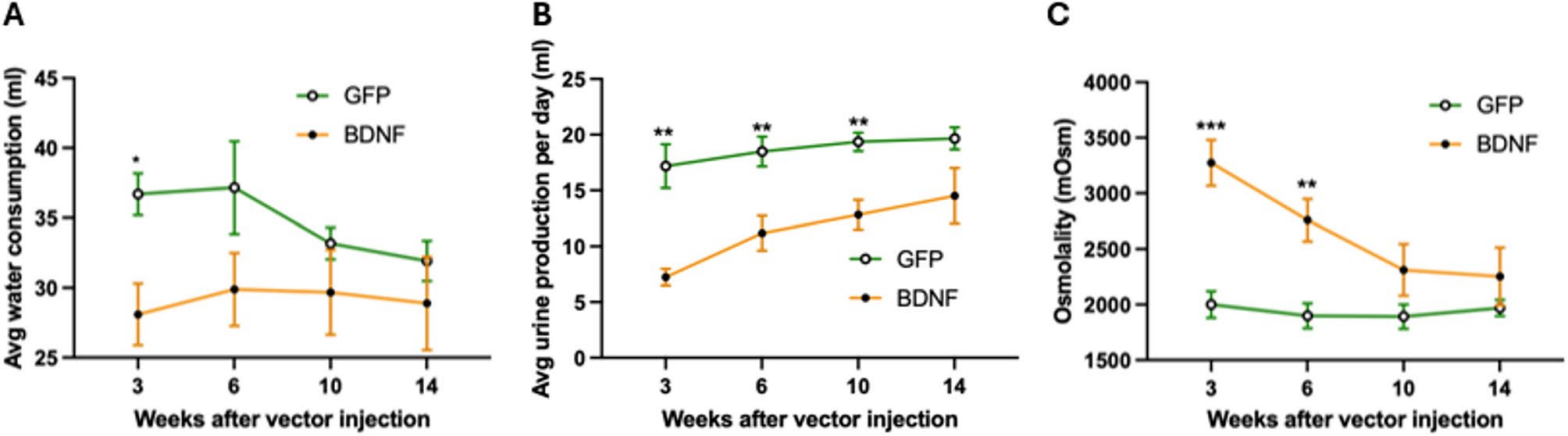
Body weight gain trajectories and verification of gene transduction in rats injected with viral vectors expressing GFP or BDNFmyc within the paraventricular nucleus of hypothalamus (PVN). **A**: Diagram indicating the location of bilateral viral vector injections into the PVN. **B**: Representative fluorescent images of coronal brain sections ~ 1.8 mm posterior to bregma showing bilateral PVN expression of GFP and unilateral or bilateral PVN expressions of BDNFmyc. 3 V: third ventricle. **C**: Body weight change of rats involved in immunohistochemical vector verifications. Weight changes from day 0 are expressed as mean ± SEM. Rats with confirmed bilateral PVN expression of BDNFmyc (n = 6) showed a markedly reduced weight gain compared to PVN-GFP controls (n = 6) over the duration of the study. Body weight trajectories from rats with unilateral PVN expression of BDNFmyc (n = 2) are colored in magenta and are not included in the overall PVN-BDNF mean. **D**: Violin plots show distribution of body weight changes for all PVN-GFP (n = 38) and PVN-BDNF animals (n = 30) included in the study as well as PVN-BDNF rats excluded (n = 10) due to missed injections. The horizontal dotted line shows our body weight change cutoff criteria (127.3 g) used to determine if animals were likely to have bilaterial or unilateral PVN-BDNF injections.

Figure 2 was omitted from the original version of the Article.

The original Fig. 2 and the accompanying legend appear below.

**Fig. 2:**
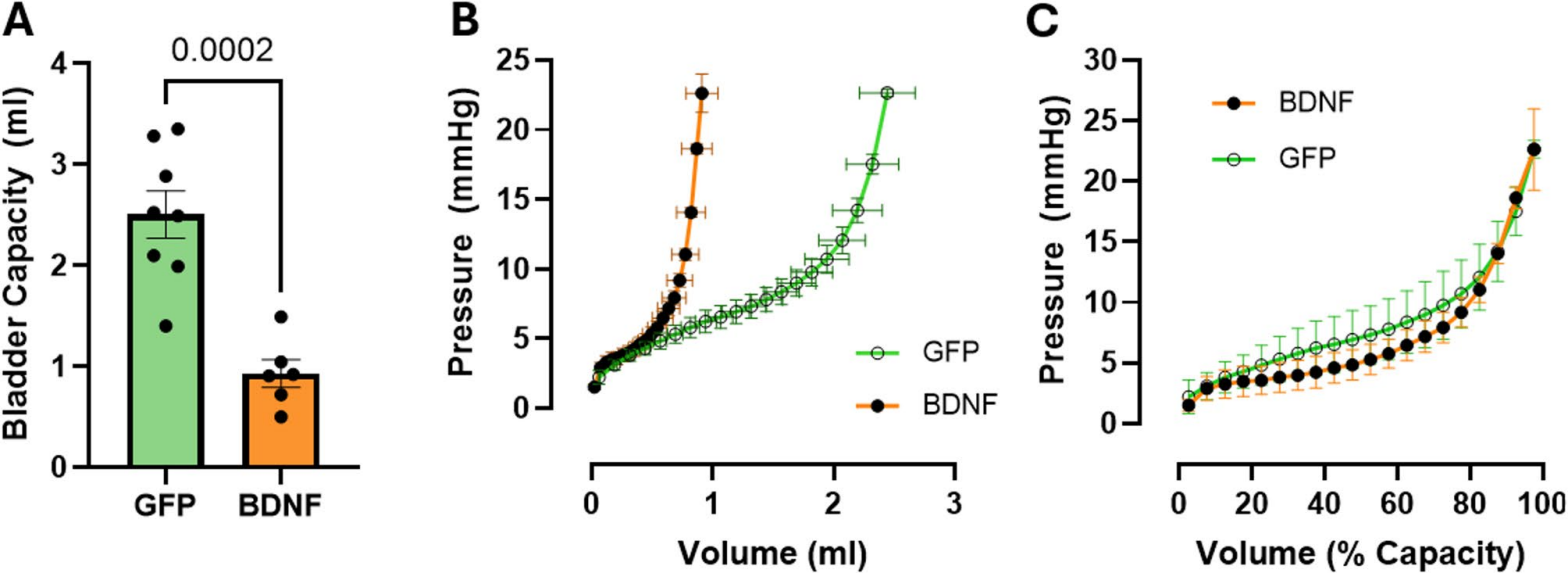
Assessment of BDNF-induced cardiovascular changes and activation of neuroendocrine stress pathways 14 to 19 weeks post viral vector injections. **A**: Cardiac left ventricle weight (LV) to body weight (BW) ratio (left, n = 4/group). **B**: Representative radiotelemetric recordings of mean arterial pressure (MAP) and heart rate (HR) averaged over 5 days in a single PVN-BDNF and a single PVN-GFP rat at 14 weeks after vector injections; shaded rectangle indicates nighttime (Lights OFF). **C**-**D**: Plasma corticosterone (GFP: n = 8; BDNF: n = 5) and AVP (GFP: n = 4; BDNF: n = 6) levels measured from blood samples collected from isoflurane-anesthetized rats using cardiac punctures before euthanasia. Results are expressed as mean ± SEM. Statistical significance was tested with unpaired t-test.

Figure 4 was a duplication of Figure 3.

The original Fig. 4 and the accompanying legend appear below.

**Fig. 4:**
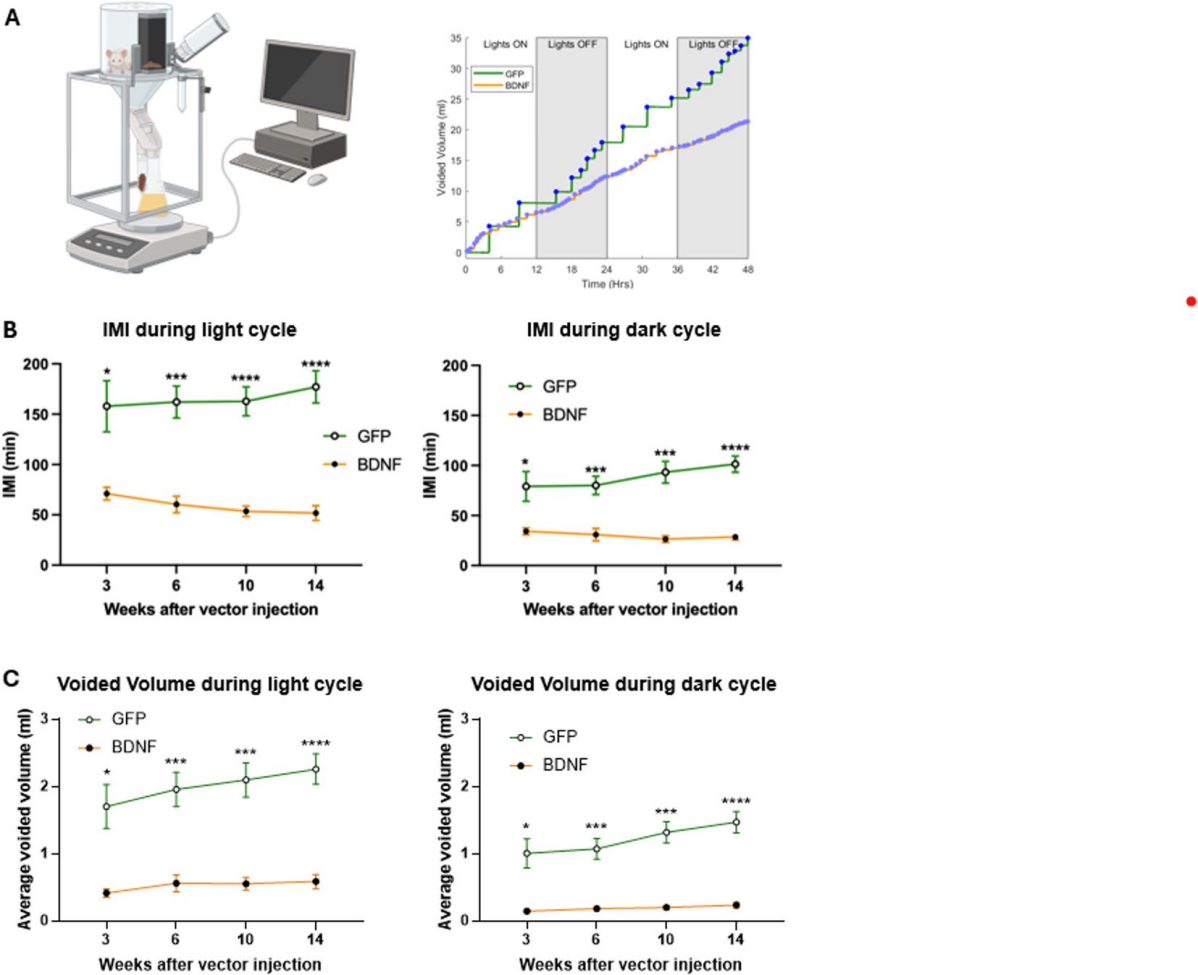
Average water consumption, urine production, and urine osmolality at weeks 3, 6, 10, and 14 following vector injections. **A**: Average daily water consumption observed at all timepoints averaged over the 48-h sessions in PVN-GFP and PVN-BDNF rats. **B**: Daily urine production at all timepoints averaged over the 48-h sessions in PVN-GFP and PVN-BDNF rats. **C**: Urine osmolality at all timepoints assessed in urine collected at the conclusion of the 48-h recording sessions in PVN-GFP and PVN-BDNF rats. Results are expressed as mean ± SEM. A Two-way repeated measures ANOVA with Tukey’s post hoc test was used. *p < 0.05, **p < 0.01 vs. control (GFP: n = 8; BDNF: n = 6).

Finally, the original Figure 6 was published as Figure 2..

The original Fig. 6 and the accompanying legend appear below.

**Fig. 6:**
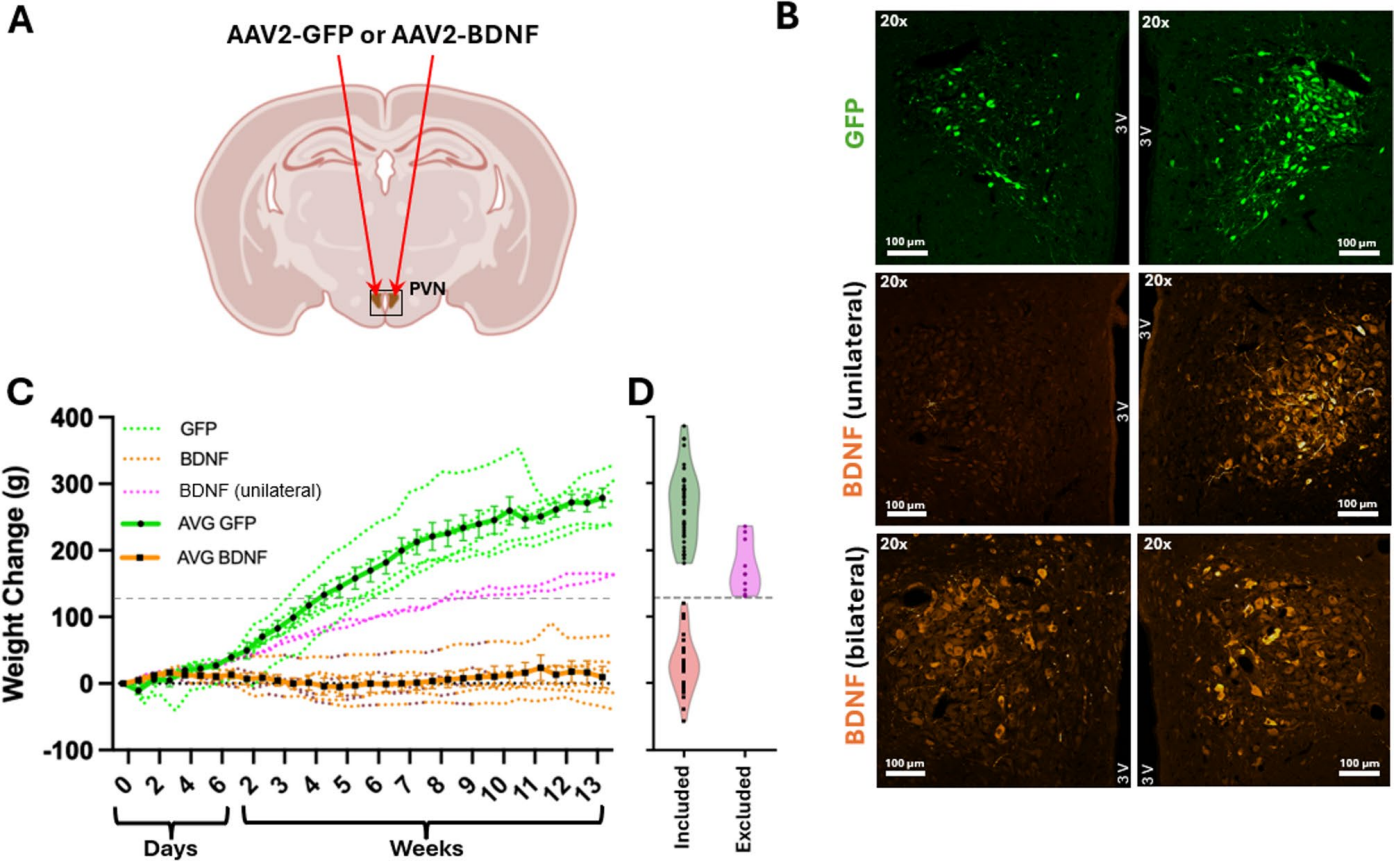
Bladder capacity and volume-pressure curves in ex vivo pressurized bladders from PVN-GFP and PVN-BDNF rats at weeks 17 to 19 after vector injections. **A**: Bladder capacity, defined as the volume a urinary bladder holds once the pressure reaches 25 mmHg. **B**: Bladder pressure vs. infused volume. **C**: Bladder pressure vs. infused volume expressed as % of bladder capacity). Unpaired t-test was used for bladder capacity and two-way repeated measures ANOVA with Tukey’s post hoc test was used for volume-pressure curves (GFP: n = 8; BDNF: n = 6). Results are expressed as mean ± SEM. P values are shown above data sets.

The original Article has been corrected.

