# Supplementary material for: Overactive bladder phenotype induced by chronic activation of hypothalamic neuroendocrine stress pathways in rats with no extrinsic behavioral stress applied
Source: Sci Rep. 2025 Dec 21;15:45068. doi: 10.1038/s41598-025-32428-6 (PMC12748808; doi:10.1038/s41598-025-32428-6)
Supplement: Supplementary file 1 — Supplementary Material 1 [file 41598_2025_32428_MOESM1_ESM.docx]

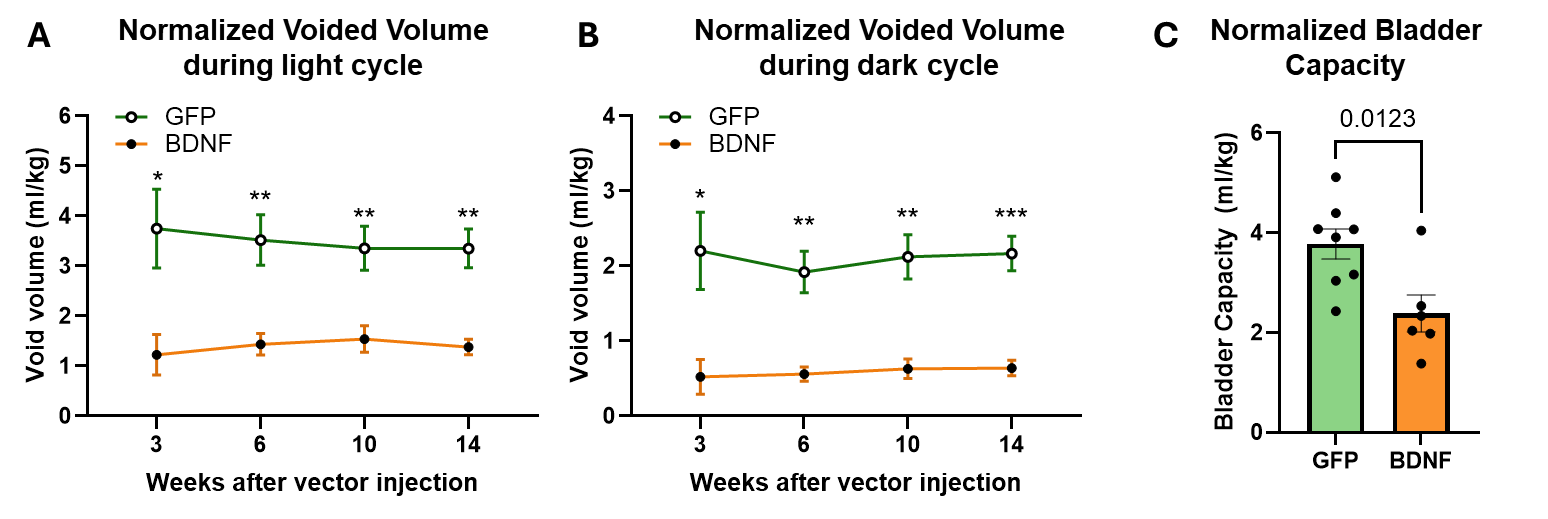


**Supplementary figure 1:** **A-B**: Noninvasive assessment of voiding behavior in PVN-GFP and PVN-BDNF rats at weeks 3, 6, 10, and 14 following vector injections. Voided volume, normalized to body weight, was significantly reduced in the PVN-BDNF group at all timepoints during both the light (left) and dark (right) phases. **C:** Bladder capacity, defined as the volume a urinary bladder holds once the pressure reaches 25 mmHg, normalized to body weight was significantly lower in the PVN-BDNF group. Results are expressed as mean ± SEM. Two-way repeated measures ANOVA with Tukey’s post hoc test was used for A-B. **p<0.05,* ***p<0.01, ***p<0.001* vs. control (GFP: *n* = 8; BDNF: *n* = 6), unpaired t-test was used for C (GFP: *n* = 8; BDNF: *n* = 6).
