# Supplementary figures and images for: Overactive bladder phenotype induced by chronic activation of hypothalamic neuroendocrine stress pathways in rats with no extrinsic behavioral stress applied
Source: Sci Rep. 2025 Dec 21;15:45068. doi: 10.1038/s41598-025-32428-6 (PMC12748808; doi:10.1038/s41598-025-32428-6)

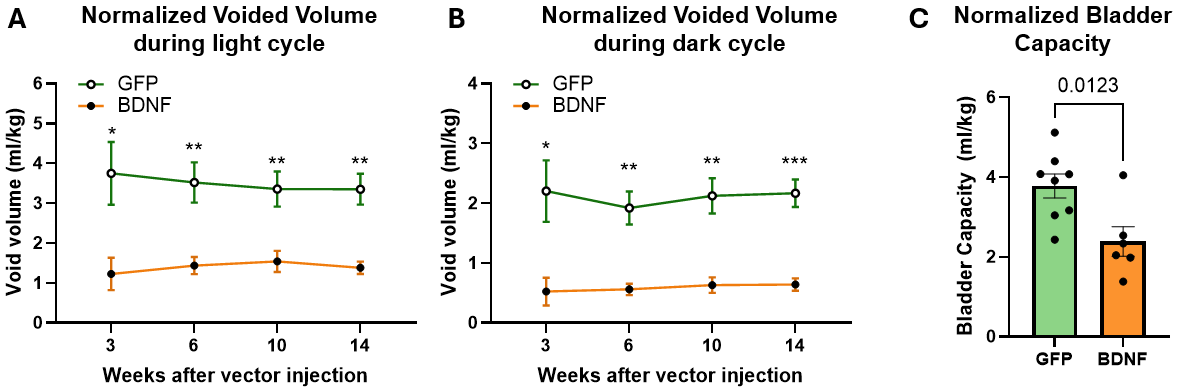

Supplement: Supplementary file 2 — Supplementary Material 2 [file 41598_2025_32428_MOESM2_ESM.png]
